# Transcatheter MitraClip repair alters mitral annular geometry – device induced annular remodeling on three-dimensional echocardiography predicts therapeutic response

**DOI:** 10.1186/s12947-019-0181-z

**Published:** 2019-12-26

**Authors:** Jiwon Kim, Maria Chiara Palumbo, Omar K. Khalique, Lisa Q. Rong, Razia Sultana, Mukund Das, Jennifer Jantz, Yasfumi Nagata, Richard B. Devereux, Shing Chiu Wong, Geoffrey W. Bergman, Robert A. Levine, Mark B. Ratcliffe, Jonathan W. Weinsaft

**Affiliations:** 1 Department of Medicine (Cardiology), Weill Cornell Medicine, 525 East 68th Street, New York, NY 10021 USA; 2 Department of Cardiothoracic Surgery, Weill Cornell Medicine, New York, NY USA; 3 Department of Anesthesiology, Weill Cornell Medicine, New York, NY USA; 4 Division of Cardiology, Columbia University Medical Center, New York, NY USA; 5 Division of Cardiology -Massachusetts General Hospital, Harvard Medical School, Boston, MA USA; 6 Department of Bioengineering, University of California, San Francisco, USA; 7 Veterans Affairs Medical Center, San Francisco, CA USA

**Keywords:** MitraClip, 3D echocardiography, Mitral annulus

## Abstract

**Background:**

Echocardiography (echo) is widely used to guide therapeutic decision-making for patients being considered for MitraClip. Relative utility of two- (2D) and three-dimensional (3D) echo predictors of MitraClip response, and impact of MitraClip on mitral annular geometry, are uncertain.

**Methods:**

The study population comprised patients with advanced (> moderate) MR undergoing MitraClip. Mitral annular geometry was quantified on pre-procedural 2D transthoracic echocardiography (TTE) and intra-procedural 3D transesophageal echocardiography (TEE); 3D TEE was used to measure MitraClip induced changes in annular geometry. Optimal MitraClip response was defined as ≤mild MR on follow-up (mean 2.7 ± 2.5 months) post-procedure TTE.

**Results:**

Eighty patients with advanced MR underwent MitraClip; 41% had optimal response (≤mild MR). Responders had smaller pre-procedural global left ventricular (LV) end-diastolic size and mitral annular diameter on 2D TTE (both *p* ≤ 0.01), paralleling smaller annular area and circumference on 3D TEE (both *p* = 0.001). Mitral annular size yielded good diagnostic performance for optimal MitraClip response (AUC 0.72, *p* < 0.01). In multivariate analysis, sub-optimal MitraClip response was independently associated with larger pre-procedural mitral annular area on 3D TEE (OR 1.93 per cm^2^/m^2^ [CI 1.19–3.13], *p* = 0.007) and global LV end-diastolic volume on 2D TTE (OR 1.29 per 10 ml/m^2^ [CI 1.02–1.63], *p* = 0.03). Substitution of 2D TTE derived mitral annular diameter for 3D TEE data demonstrated a lesser association between pre-procedural annular size (OR 5.36 per cm/m^2^ [CI 0.95–30.19], *p* = 0.06) and sub-optimal MitraClip response. Matched pre- and post-procedural TEE analyses demonstrated MitraClip to acutely decrease mitral annular area and circumference (all *p* < 0.001) as well as mitral tenting height, area, and volume (all *p* < 0.05): Magnitude of MitraClip induced reductions in mitral annular circumference on intra-procedural 3D TEE was greater among patients with, compared to those without, sub-optimal MitraClip response (>mild MR) on followup TTE (*p* = 0.017); greater magnitude of device-induced annular reduction remained associated with sub-optimal MitraClip response even when normalized for pre-procedure annular circumference (*p* = 0.028).

**Conclusions:**

MitraClip alters mitral annular geometry as quantified by intra-procedural 3D TEE. Pre-procedural mitral annular dilation and magnitude of device-induced reduction in mitral annular size on 3D TEE are each associated with sub-optimal therapeutic response to MitraClip.

## Background

Echocardiography (echo) is widely used to diagnose mitral regurgitation (MR) and assess its response to therapeutic interventions. MitraClip is the sole percutaneous device commercially approved in the United States to treat MR, and is increasingly utilized worldwide for this purpose – over 80,000 patients have undergone MitraClip in the last decade [1]. Recurrent MR after MitraClip is a substantial problem. Studies have reported residual or recurrent (> mild) MR in over one third of patients undergoing MitraClip [2–5]. Even when implanted in controlled research settings, MitraClip response varies – as evidenced by two recent trials (MITRA-FR, COAPT) that yielded conflicting results regarding impact of MitraClip on MR reduction and clinical outcomes [6, 7].

One reason for variable MR response to MitraClip stems from patient-specific differences in cardiac chamber remodeling. MitraClip is intended to reduce MR via focal leaflet coaptation. However, prior echo studies by our group and others have shown increased left ventricular (LV) size to augment risk for recurrent MR after MitraClip implantation [5], supporting the notion that remodeling indices beyond mitral valve anatomy impact therapeutic response. It is also possible that the device itself contributes to risk for MR recurrence. MitraClip induced leaflet tension may alter annular geometry – thus contributing to recurrent MR via distortion of valve coaptation and tethering of peri-annular myocardium. Consistent with this, computational modeling studies have shown MitraClip to augment leaflet stress adjacent to the device and also to affect broader aspects of the mitral apparatus - including decreased annular size and increased stretch (displacement) of peri-annular LV myocardium [8]. It remains unclear whether clinical application of MitraClip produces in vivo alterations in mitral annular geometry, and how such remodeling impacts patient outcomes.

This study tested impact of MitraClip on mitral annular remodeling. To do so, intra-procedural three-dimensional (3D) transesophageal echo (TEE) was used to quantify annular geometry prior to and immediately after MitraClip implantation. Transthoracic echo (TTE) was analyzed pre- and early (within 6 months) post-procedure to evaluate change in MR. Study goals were to test whether MitraClip acutely alters mitral annular geometry in a manner discernable via 3D TEE, and if magnitude of device-induced alterations in annular geometry stratifies MitraClip therapeutic response.

## Methods

The study population comprised consecutive patients with advanced (>moderate) MR who underwent MitraClip at Weill Cornell Medicine (NY, NY) in whom intraprocedural TEE was available to evaluate annular geometry, and TTE was performed pre- and post- (1–6 months [target 6 months]) procedure to assess change in MR: No otherwise eligible patients were excluded based on procedural outcomes, imaging findings, or clinical indices.

Demographic data were collected in a standardized manner, including cardiac risk factors and medications. Procedural indices including number of MitraClip devices were also recorded. Ambulatory blood pressure, heart rate, and cardiac rhythm was measured at time of baseline and followup TTE. This analysis of pre-existing (retrospective) data for research purposes was approved by the Weill Cornell Institutional Review Board.

### Image acquisition

To evaluate mitral annular and cardiac chamber geometry in relation to MitraClip response, data were derived from TEE and TTE, both of which were acquired via a standardized protocol:

- TEEs were acquired intra-procedure in a mid-esophageal view using Philips iE33 or EPIQ7 systems equipped with matrix array transducers. 3D images of the mitral apparatus including the annulus were optimized for coverage and gain using single beat acquisition (Zoom 3D); datasets were selected for analysis based on optimal discernment of the mitral annulus.
- TTEs were obtained using commercial equipment. Images were acquired in parasternal long, parasternal short, and apical 2-, 3-, and 4- chamber orientations. Color and pulsed wave Doppler was used to assess MR; continuous wave Doppler included assessment of tricuspid regurgitant velocity (to quantify pulmonary artery [PA] systolic pressure).

### Image analysis

#### Three dimensional mitral annular geometry

**Fig. 1:**
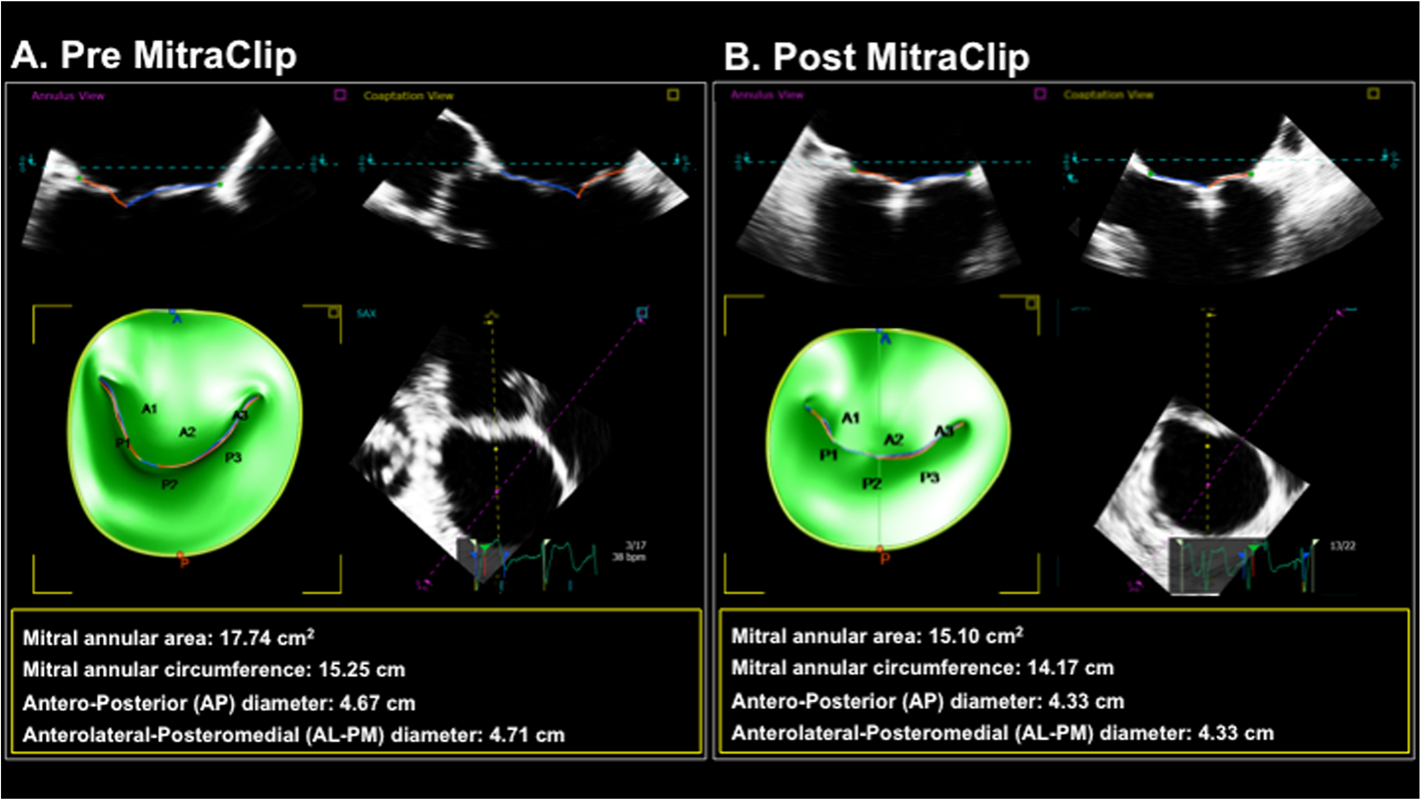
MitraClip Induced Mitral Annular Remodeling on 3D Transesophageal Echo. Representative example of mitral apparatus remodeling parameters as quantified using intra-procedural 3D TEE. Note device-induced reductions in mitral annular circumference and area, paralleling post-procedure reductions in mitral annular linear indices

3D TEE analysis was performed using a semi-automated program (TomTEC 4D MV [Munich, Germany]) tailored for 3D mitral annular modeling. All mesurements were performed blinded to MitraClip response. A late-systolic frame was selected for annular tracking defined as the last systolic frame before mitral valve opening. Landmarks denoting anterior and posterior mitral annulus were identified on long orthogonal views followed by identification of the coaptation points of the aortic and mitral valves and apical posterior aspect of the aortic annulus. Semi-automated contours of the mitral annulus and mitral valve leaflets were then generated throughout the cardiac cycle. Contours were were manually edited via rotation around the mitral annulus to ensure accurate border segmentation. Calculated indices included valvular tenting area and height, mitral annular linear (antero-posterior, anterolateral-posteromedial) dimensions, as well as mitral annular area and circumference (see Fig. 1 for representative example).

#### Chamber quantification

LV chamber size, function, and mass were quantified on TTE based on linear dimensions in parasternal long axis orientation, concordant with established methods validated in prior research [9, 10]. LV end-diastolic and end-systolic internal dimensions were measured at the level of mitral leaflet tips; mitral annular diameter was measured as the distance between annular insertion into the lateral LV wall and inferoseptum at LV end-diastole in apical 4-chamber orientation. LV mass was quantified using anteroseptal and posterior wall thickness, concordant with validated methods [11]. LV global longitudinal strain was calculated based on aggregate (2, 3, 4-chamber) long axis data in accordance with established methods previously applied by our group [12]. Left atrial (LA) area was quantified in apical 2- and 4-chamber orientation, for which results were used to quantify LA volume. LA global longitudinal and circumferential strain was quantified 4 chamber orientation; strain indices were derived using commercial software (TomTEC [Munich, Germany]), for which automated border detection was manually adjusted to ensure optimal tracking throughout the cardiac cycle.

#### Mitral regurgitation

MR severity was analyzed on TTE by dedicated ACC/AHA level III trained readers in a high-volume laboratory, for which expertise in MR quantification has been documented [13, 14]. MR was graded for study purposes using consensus guidelines based on an aggregate 4-point scale (1 = mild – 4 = severe) [15], for which primary components included vena contracta, regurgitant fraction, regurgitant volume, and effective regurgitant orifice area (EROA).

Optimal MitraClip response was defined as ≤mild MR on follow-up TTE; a criterion concordant with prior surgical mitral repair literature [16], as well as MitraClip outcomes studies [3, 4].

### Statistical methods

Comparisons between groups were made using Student’s t test (expressed as mean ± standard deviation) for continuous variables, and chi-square test for categorical variables. Bivariate correlation coefficients were used to evaluate associations between continuous variables. Univariable and multivariate modeling was performed via binary logistic regression. Diagnostic utility of remodeling indices was evaluated in relation to MitraClip response using receiver operator characteristics curves, for which area under the curve (AUC) was used as an index of overall test performance. Statistical calculations were performed using SPSS 22.0 (SPSS Inc. [Chicago, IL]). Two-sided *p* < 0.05 was considered indicative of statistical significance.

## Results

### Population characteristics

The study population comprised 80 patients with advanced (> moderate) MR who underwent MitraClip as well as pre- and post-procedure TTE to assess procedural durability, reflecting 78% of all patients who underwent this procedure at our site (Weill Cornell Medicine [NY, NY]) during the study interval (2013–19). MitraClip implantation employed the NTr device type in over three-fourths of cases (87% NT/NTr; 13% XTr). In nearly all cases (76/80), MR was deemed primarily degenerative; leading pathologies were prolapse, annular calcification, and valve thickening. However, concommitant conditions predisposing to mixed MR (with functional component) was common; 26% of patients had prior MI and 47% had systolic heart failure (LVEF< 50%). All patients underwent MitraClip without complications; a mean of 1.63 ± 0.58 devices were implanted per procedure (58% had multiple devices implanted during the index intervention).

Follow-up TTE (2.7 ± 2.5 months) was performed to assess short term procedural response based on MR: Whereas nearly all (91%) patients had some improvement in MR (≥1 grade MR reduction), less than half (41%) had optimal MitraClip response (≤mild MR). Regarding thereapeutic response, follow-up data demonstrated MR trace/absent in 10% (33% 1+ [mild] / 17% 2+ [moderate]/ 30% 3+ [moderately-severe] / 10% 4+ [severe]). Table 1 reports clinical characteristics over the overall study population, including comparisons between patients with and without optimal MitraClip response (≤ mild [1+] MR). As shown, CAD and associated clinical risk factors for adverse LV chamber remodeling were highly common, but of similar prevalence between groups stratified based on MitraClip response (all *p* = NS).

**Table 1:** Clinical characteristics

	Overall (*n* = 80)	MC_RSP_ + ^a^ (*n* = 33)	MC_RSP_ – (*n* = 47)	p
Age (year)	79 ± 10	79 ± 9	80 ± 10	0.72
Male gender	63% (50)	64% (21)	62% (29)	0.86
Heart rate (bpm)	70 ± 12	72 ± 14	70 ± 11	0.52
Systolic blood pressure (mmHg)	116 ± 18	119 ± 18	113 ± 17	0.17
Diastolic blood pressure (mmHg)	65 ± 12	64 ± 13	66 ± 10	0.59
Atherosclerosis Risk Factors				
Hypertension	81% (65)	88% (29)	77% (36)	0.20
Hypercholesterolemia	66% (53)	61% (20)	70% (33)	0.37
Diabetes mellitus	29% (23)	39% (13)	21% (10)	0.08
Tobacco use	61% (49)	55% (18)	66% (31)	0.30
Coronary Artery Disease	54% (43)	52% (17)	55% (26)	0.74
Prior Myocardial Infarction	26% (21)	21% (7)	30% (14)	0.39
Prior Revascularization	41% (33)	39% (13)	43% (20)	0.78
Atrial fibrillation/flutter	39% (31)	39% (13)	38% (18)	0.92
Cardiovascular Medications				
Beta-blocker	79% (63)	82% (27)	77% (36)	0.57
ACE-Inhibitor/Angiotensin Receptor Blocker	56% (45)	52% (17)	60% (28)	0.47
Loop diuretic	81% (65)	85% (28)	79% (37)	0.49
HMG CoA-Reductase Inhibitor	69% (55)	70% (23)	68% (32)	0.88
Aspirin	59% (47)	52% (17)	64% (30)	0.27
Number of Clips Implanted				
Mean	1.63 ± 0.58	1.61 ± 0.50	1.64 ± 0.64	0.81
Multiple (> 1)	46 (57%)	20 (61%)	26 (55%)	0.64

^a^Optimal MC_RSP_ defined as ≤ mild MR after MitraClip

### MitraClip response in relation to chamber geometry

Table 2 reports 2D TTE derived structural and functional parameters in relation to MitraClip response. Despite similar clinical profiles, results demonstrate that MitraClip non-responders had more advanced chamber remodeling, as evidenced by increased mitral annular diameter and global LV size (both *p* < 0.01). Regarding LA remodeling, results similarly demonstrated sub-optimal MitraClip to be associated with chamber dilation: LA area was, on average, more than 25% larger among MitraClip non-responders – results were similar based on data acquired in 2- and 4-chamber orientation (both *p* < 0.05), as well as calculated LA volume (*p* = 0.01). LA dilation was accompanied by impaired LA function; LA circumferential and longitundinal strain were both lower among MitraClip non-responders (*p* < 0.05): circumferential (*r* = − 0.47) and longitudinal strain (*r* = − 0.35, both *p* < 0.001) were each negatively correlated with LA area.

**Table 2:** Baseline imaging characteristics

	Overall (*n* = 80)	MC_RSP_ + ^a^ (*n* = 33)	MC_RSP_ – (*n* = 47)	p
Mitral Regurgitation				
*Regurgitant Severity*				
Regurgitant fraction (%)	56 ± 16	53 ± 12	58 ± 19	0.19
EROA (cm^2^)	0.6 ± 0.3	0.5 ± 0.3	0.6 ± 0.3	0.18
Regurgitant volume (ml)	88 ± 39	80 ± 35	94 ± 41	0.13
*Mitral Valve Morphology*				
Prolapse	37% (30)	39% (13)	36% (17)	0.77
Mitral annular calcification	41% (33)	45% (15)	38% (18)	0.52
Mitral valve thickening	57% (46)	55% (18)	60% (28)	0.65
Flail pathology	14% (11)	12% (4)	15% (7)	1.00
Left Ventricle				
Ejection fraction (%)	49 ± 15	48 ± 15	49 ± 15	0.99
Global Longitudinal Strain (%)	15.8 ± 5.3	16.1 ± 4.8	15.3 ± 5.7	0.48
End-diastolic diameter (cm)	6.0 ± 0.8	5.8 ± 0.7	6.2 ± 0.8	**0.007**
End-diastolic volume (ml/m^2^)	104 ± 30	94 ± 25	111 ± 31	**0.01**
End-systolic diameter (cm)	4.5 ± 1.1	4.3 ± 1.0	4.7 ± 1.1	0.10
End-systolic volume (ml/m^2^)	56 ± 32	49 ± 27	61 ± 34	0.12
Mitral Annular Diameter	3.36 ± 0.49	3.19 ± 0.45	3.48 ± 0.49	**0.009**
Myocardial mass (g/m^2^)	112 ± 31	107 ± 28	116 ± 33	0.21
Relative wall thickness	0.26 ± 0.05	0.28 ± 0.05	0.25 ± 0.05	**0.02**
Left Atrium				
Diameter (cm)	5.0 ± 1.1	4.8 ± 1.0	5.2 ± 1.1	0.10
2-Chamber area (cm^2^)	32 ± 12	29.0 ± 7.6	34.5 ± 13.3	**0.03**
4-Chamber area (cm^2^)	33 ± 10	28.6 ± 7.4	35.4 ± 10.8	**0.003**
Volume (ml/m^2^)	73 ± 37	62 ± 27	82 ± 41	**0.01**
Global Circumferential Strain (%)	13.4 ± 7.2	15.5 ± 7.6	12.0 ± 6.6	**0.03**
Global Longitudinal Strain (%)	12.5 ± 5.1	14.1 ± 5.7	11.5 ± 4.3	**0.02**
Ejection Fraction (%)	35 ± 15	37 ± 15	33 ± 16	0.19
Right Ventricle				
TAPSE (cm)	1.8 ± 0.6	1.8 ± 0.5	1.8 ± 0.6	0.55
S′ (cm/s)	10.7 ± 3.0	10.3 ± 2.7	11.1 ± 3.2	0.23
Pulmonary artery systolic pressure (mmHg)	52 ± 16	55 ± 18	51 ± 14	0.21

^a^Optimal MC_RSP_ defined as ≤ mild MR after MitraClip

Data in bold are significant

Table 3 reports 3D mitral apparatus remodeling indices as quantified on TEE (available in 83% [*n* = 66]). As shown, 3D TEE results paralleled those of 2D TTE with respect to associations between adverse remodeling and MitraClip response: Mitral annular area, circumference, and diameter were all equivalently larger among patients with suboptimal MitraClip response (*p* < 0.001), paralleling a trend towards increased tenting height (*p* = 0.08) in sub-optimal responders.

**Table 3:** Pre-procedural transesophageal echo mitral annular indices relation to MitraClip response

	MC_RSP_ + ^a^ (*n* = 26)	MC_RSP_ – (*n* = 40)	p
Mitral Annular Area (cm^2^)	12.85 ± 1.90	15.28 ± 3.48	**0.001**
(cm^2^/m^2^)	7.07 ± 1.02	8.49 ± 2.06	**< 0.001**
Annular Circumference (cm)	13.10 ± 0.97	14.27 ± 1.54	**0.001**
(cm/m^2^)	7.23 ± 0.83	7.95 ± 1.25	**0.01**
Antero-Posterior Diameter (cm)	3.76 ± 0.36	4.12 ± 0.53	**0.003**
Anterolateral-Posteromedial Diameter (cm)	4.20 ± 0.38	4.54 ± 0.51	**0.005**
Annular Height (cm)	0.72 ± 0.17	0.82 ± 0.24	0.06
Nonplanar Angle (°)	154.05 ± 9.98	150.06 ± 12.40	0.17
Tenting Volume (cm^3^)	3.54 ± 1.86	4.22 ± 1.70	0.14
Tenting Area (cm^2^)	1.80 ± 0.78	2.09 ± 0.75	0.13
Tenting Height (mm)	8.63 ± 3.06	9.92 ± 2.79	0.08
Anterior Leaflet Angle (°)	36.53 ± 14.86	35.05 ± 13.49	0.68
Posterior Leaflet Angle (°)	18.97 ± 6.45	21.90 ± 7.39	0.10

^a^Optimal MC_RSP_ defined as ≤ mild MR after MitraClip

Data in bold are significant

### Remodeling based predictors of MitraClip response

Regression analyses were used to test relative utility of remodeling indices for prediction of MitraClip response. As shown in Table 4, univariate analyses demonstrated likelihood of optimal response (≤mild MR) at follow-up to decrease in relation to magnitude of increased mitral annular size on pre-procedural TEE, irrespective of whether quantified based on annular area (OR 1.93 per cm^2^/m^2^ [CI 1.22–3.06]), circumference (OR 1.96 per cm/m^2^ [CI 1.12–3.41]), or corresponding 2D area and linear based indices (all *p* < 0.05): Predictive utility of TEE indices of mitral annular size were similar, as evidenced by similar overall diagnostic performance (AUC 0.65–0.72). Table 4 also demonstrates TTE derived cardiac chamber remodeling indices to be associated with MitraClip response: LV (OR 1.25 per 10 ml/m^2^ [CI 1.04–1.50]) and LA (OR 1.22 per 10 ml/m^2^ [CI 1.03–1.44]) volume were each associated (*p* < 0.05) with greater residual MR (>mild) after MitraClip implantation. Similarly, increased mitral annular diameter as quantified on TTE was associated with MitraClip response (*p* = 0.02), as was the case for annular size on intra-oprocedural TEE imaging (*p* < 0.01).

**Table 4:** Structural predictors of sub-optimal MitraClip response

	Odds Ratio (95% Confidence Interval)	p	AUC	Cutoff^b^	Sensitivity	Specificity
*3D Transesophogeal Echo*						
Mitral Annular Area (cm^2^/m^2^)	1.93 (1.22–3.06)	**0.005**	0.72 (0.59–0.84)	6.72	82%	46%
Annular Circumference (cm/m^2^*)*	1.96 (1.12–3.41)	**0.02**	0.65 (0.52–0.79)	6.83	82%	42%
Antero-Posterior Diameter (cm)	7.16 (1.72–29.85)	**0.007**	0.72 (0.60–0.85)	3.77	80%	54%
Anterolateral-Posteromedial Diameter (cm)	6.35 (1.58–25.44)	**0.009**	0.70 (0.57–0.82)	4.03	87%	38%
Annular Height (cm)	8.27 (0.73–93.28)	0.09	0.63 (0.50–0.77)	0.62	82%	35%
Tenting Volume (cm^3^)	1.26 (0.93–1.70)	0.14	0.64 (0.50–0.78)	2.69	82%	38%
Tenting Area (cm^2^)	1.70 (0.85–3.38)	0.13	0.63 (0.49–0.77)	1.51	80%	38%
Tenting Height (mm)	1.17 (0.98–1.40)	0.09	0.64 (0.50–0.79)	7.80	87%	50%
Anterior Leaflet Angle (°)	0.99 (0.96–1.03)	0.67	0.50 (0.35–0.64)	24.55	82%	27%
Posterior Leaflet Angle (°)	1.06 (0.99–1.15)	0.11	0.64 (0.50–0.79)	16.05	85%	46%
Nonplanar Angle (°)	0.97 (0.93–1.01)	0.17	0.40 (0.26–0.54)	140.40	82%	11%
*2D Transthoracic Echo*						
Mitral Annular Diameter *(*cm/m^2^*)*	6.50 (1.27–33.20)	**0.02**	0.64 (0.51–0.76)	1.67	81%	46%
LV End-diastolic volume (ml/m^2^)^a^	1.25 (1.04–1.50)	**0.01**	0.67 (0.55–0.79)	8.46	81%	39%
LA Volume (ml/m^2^)^a^	1.22 (1.03–1.44)	**0.02**	0.68 (0.56–0.80)	5.41	83%	54%
LA 2-Chamber area (cm^2^)	1.06 (1.00–1.11)	**0.04**	0.64 (0.52–0.77)	24.65	87%	33%
LA 4-Chamber area (cm^2^)	1.09 (1.02–1.16)	**0.006**	0.68 (0.56–0.80)	27.95	81%	54%

^a^per 10 ml increment

^b^cutoffs chosen for maximum specificity with minimum sensitivity ≥80%

Data in bold are significant

Multivariate analysis was used to further test the association of pre-procedural remodeling indices with MitraClip response. As shown in Table 5A and B, sub-optimal MitraClip response was independently associated with increased mitral annular area on 3D TEE (OR 1.93 per cm^2^/m^2^ [CI 1.19–3.13], *p* = 0.007) as well as global LV volume as quantified by 2D TTE (OR 1.29 per 10 ml/m^2^ [CI 1.02–1.63], *p* = 0.03). Substitution of 2D TTE derived mitral annular diameter (in place of corresponding 3D TEE area) weakened the predictive model (χ^2^ 17.22 ➔ 10.87), and demonstrated a lesser association between pre-procedural annular diameter (*p* = 0.06) and greater residual MR (>mild) after MitraClip implantation.

**Table 5:** Multivariate models for prediction of sub-optimal MitraClip response

5A.	Multivariate Regression *Model chi square = 17.22, p < 0.001*	
Variable	Odds Ratio (95% Confidence Interval)	p
Mitral Annular Area (cm^2^/m^2^)	1.93 (1.19–3.13)	**0.007**
LV End Diastolic Volume (ml/m^2^)^a^	1.29 (1.02–1.63)	**0.03**
5B.	Multivariate Regression *Model chi square = 10.87, p = 0.004*	
Variable	Odds Ratio (95% Confidence Interval)	p
Mitral Annular Diameter (cm/m^2^)	5.36 (0.95–30.19)	0.06
LV End Diastolic Volume (ml/m^2^)^a^	1.23 (1.02–1.49)	**0.03**

^a^per 10 ml increment

Data in bold are significant

### MitraClip induced annular remodeling

Given our observed association between pre-procedural mitral annular area and therapeutic response, 3D TEE data were further analyzed to test whether MitraClip acutely altered annular geometry, and whether such alterations predicted procedural success. Table 6 reports pre- and post-procedural mitral annular geometry among the overall cohort, demonstrating device-induced reductions in mitral annular area and circumference, as well as valvular tenting area and volume (all *p* < 0.001): Fig. 1 provides a representative example of MitraClip induced alterations in annular geometry. Of note, Table 6 also demonstrates that MitraClip induced changes in annular geometry (area, circumferential, and linear indices) generally were similar among patient subgroups with mitral prolapse (*n* = 19) and prominent annular calcification (*n* = 25) – although device-induced reductions in tenting indices were non-significant in patients with prolapse.

**Table 6:** MitraClip induced annular remodeling

	Pre	Post	Δ	p
*Overall*^a^				
Mitral Annular Area (cm^2^)	14.20 ± 2.97	12.88 ± 2.80	−1.31 ± 1.20	**< 0.001**
(cm/m^2^)	7.75 ± 1.60	7.02 ± 1.42	− 0.73 ± 0.65	**< 0.001**
Annular Circumference (cm)	13.73 ± 1.36	13.15 ± 1.35	− 0.57 ± 0.58	**< 0.001**
(cm/m^2^)	7.53 ± 1.07	7.21 ± 0.98	− 0.32 ± 0.32	**< 0.001**
Antero-Posterior Diameter (cm)	3.96 ± 0.44	3.61 ± 0.42	− 0.35 ± 0.25	**< 0.001**
Anterolateral-Posteromedial Diameter (cm)	4.37 ± 0.48	4.32 ± 0.48	− 0.05 ± 0.29	0.20
Annular Height (cm)	0.77 ± 0.21	0.71 ± 0.20	− 0.06 ± 0.21	0.06
Tenting Volume (cm^3^)	4.07 ± 1.90	3.35 ± 1.58	− 0.72 ± 0.95	**< 0.001**
Tenting Area (cm^2^)	2.01 ± 0.84	1.71 ± 0.65	− 0.29 ± 0.57	**0.001**
Tenting Height (mm)	9.44 ± 3.17	8.58 ± 2.51	− 0.86 ± 2.45	**0.015**
Anterior Leaflet Angle (°)	36.12 ± 13.88	34.32 ± 13.86	− 1.80 ± 9.96	0.20
Posterior Leaflet Angle (°)	21.05 ± 7.79	22.06 ± 6.61	1.02 ± 6.59	0.27
Nonplanar Angle (°)	151.60 ± 11.99	148.03 ± 15.46	−3.57 ± 14.67	0.09
*Mitral Prolapse*				
Mitral Annular Area (cm^2^)	14.37 ± 3.26	13.06 ± 3.40	− 1.31 ± 0.96	**< 0.001**
(cm/m^2^)	7.77 ± 1.72	7.04 ± 1.66	− 0.73 ± 0.58	**< 0.001**
Annular Circumference (cm)	13.84 ± 1.45	13.29 ± 1.54	− 0.55 ± 0.48	**< 0.001**
(cm/m^2^)	7.52 ± 1.12	7.21 ± 1.00	− 0.31 ± 0.29	**< 0.001**
Antero-Posterior Diameter (cm)	3.94 ± 0.46	3.57 ± 0.49	− 0.37 ± 0.27	**< 0.001**
Anterolateral-Posteromedial Diameter (cm)	4.40 ± 0.55	4.38 ± 0.57	− 0.02 ± 0.28	0.77
Annular Height (cm)	0.79 ± 0.22	0.72 ± 0.19	−0.07 ± 0.18	0.13
Tenting Volume (cm^3^)	2.59 ± 1.02	2.32 ± 1.27	−0.27 ± 0.86	0.22
Tenting Area (cm^2^)	1.38 ± 0.60	1.32 ± 0.57	−0.06 ± 0.62	0.69
Tenting Height (mm)	7.53 ± 3.19	7.48 ± 2.55	−0.05 ± 2.82	0.94
Anterior Leaflet Angle (°)	25.66 ± 10.16	27.19 ± 11.58	1.54 ± 10.47	0.53
Posterior Leaflet Angle (°)	20.24 ± 10.11	21.85 ± 8.65	1.61 ± 8.31	0.41
Nonplanar Angle (°)	149.44 ± 13.64	144.52 ± 17.69	− 4.92 ± 16.00	0.20
*Mitral Annular Calcification*				
Mitral Annular Area (cm^2^)	15.05 ± 3.57	13.58 ± 3.29	−1.47 ± 1.33	**< 0.001**
(cm/m^2^)	8.28 ± 1.82	7.45 ± 1.61	−0.82 ± 0.74	**< 0.001**
Annular Circumference (cm)	14.11 ± 1.58	13.48 ± 1.49	−0.63 ± 0.58	**< 0.001**
(cm/m^2^)	7.81 ± 1.18	7.46 ± 1.06	−0.35 ± 0.33	**< 0.001**
Antero-Posterior Diameter (cm)	4.08 ± 0.50	3.75 ± 0.46	−0.33 ± 0.26	**< 0.001**
Anterolateral-Posteromedial Diameter (cm)	4.49 ± 0.59	4.36 ± 0.56	−0.12 ± 0.27	**0.03**
Annular Height (cm)	0.79 ± 0.20	0.73 ± 0.22	−0.07 ± 0.22	0.12
Tenting Volume (cm^3^)	4.23 ± 1.99	3.46 ± 1.61	−0.77 ± 0.93	**0.001**
Tenting Area (cm^2^)	1.97 ± 0.91	1.72 ± 0.65	−0.25 ± 0.62	0.055
Tenting Height (mm)	8.86 ± 3.42	8.38 ± 2.69	−0.48 ± 2.67	0.38
Anterior Leaflet Angle (°)	31.70 ± 12.63	31.41 ± 12.22	−0.30 ± 9.88	0.88
Posterior Leaflet Angle (°)	20.08 ± 8.88	21.62 ± 7.63	1.54 ± 7.62	0.32
Nonplanar Angle (°)	150.67 ± 11.12	146.64 ± 16.80	− 4.03 ± 14.20	0.17

^a^pre and post-procedural 3D TEE data available in 51 patients (77% of patients with TEE)

Data in bold are significant

**Fig. 2:**
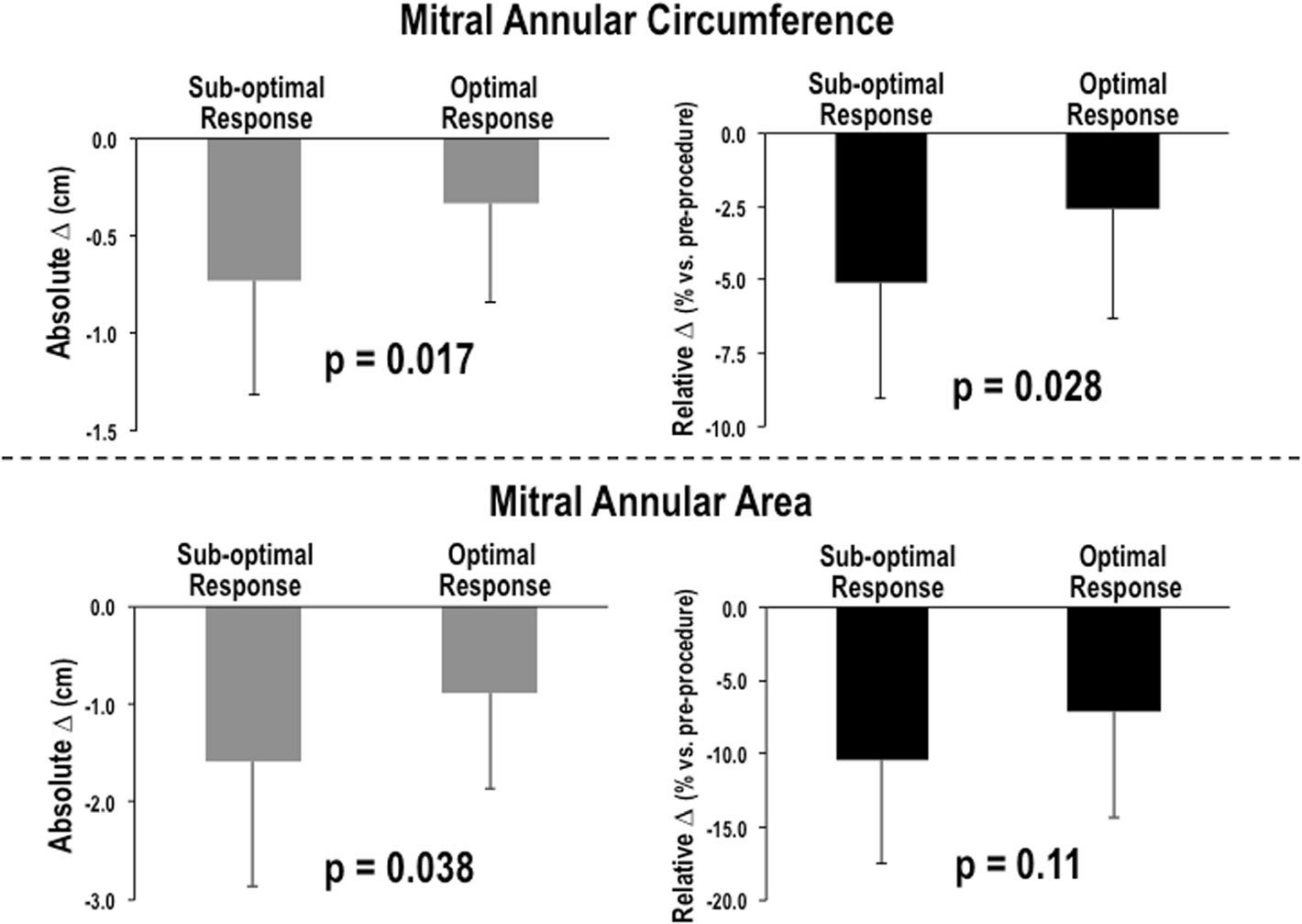
MitraClip Induced Annular Remodeling in Relation to MR Response. Magnitude of acute MitraClip induced reduction in mitral annular circumference (top) and area (bottom), as quantified using intra-procedural TEE, among patients stratified based on optimal MitraClip response (≤mild MR) as assessed on followup ambulatory TTE. Left: Greater magnitude of device-induced annular remodeling (reduced size) was observed among patients with sub-optimal MitraClip response, irrespective of whether quantified based on absolute change (pre – post) in 3D annular circumference or area (both *p* < 0.05). Right: Similar results were obtained when examining relative change ([pre – post] / pre) in annular remodeling indices. Data shown as mean ± standard deviation

As illustrated in Fig. 2, magnitude of MitraClip induced reductions in annular circumference on intra-procedural TEE was nearly 2-fold greater among patients with, compared to those without, sub-optimal MitraClip response (> mild MR) on followup TTE (0.73 ± 0.58 vs. 0.34 ± 0.50 cm, *p* = 0.017); greater magnitude of MitraClip induced annular reduction remained significantly associated with MitraClip response even when normalized for pre-procedural circumference (*p* = 0.028). Similarly, 3D TEE analysis demonstrated intra-procedural device-induced reductions in mitral annular area to be greater among patients with sub-optimal MitraClip response (1.59 ± 1.26 vs. 0.89 ± 0.98 cm^2^, *p* = 0.038) on follow-up TTE.

## Discussion

This is the first study to examine the impact of MitraClip on mitral annular remodeling - findings provide new insights regarding mechanistic determinants of therapeutic response, as well as novel echo-based parameters to guide procedural decision-making. Results demonstrate that MitraClip alters annular geometry in regions beyond the device itself, as evidenced by acute device-induced reductions in annular circumference and area (*p* < 0.001) as quantified on intra-procedural 3D TEE. Consistent with the notion that remodeling factors beyond the device impact device efficacy, pre-procedural mitral annular and LV chamber size each independently predicted sub-optimal MitraClip response; substitution of 3D TEE derived mitral annular area for annular diameter on 2D TTE strengthened the predictive model. Magnitude of intra-procedural device-induced reduction in mitral annular size was greater among patients with sub-optimal MitraClip response (>mild MR) on followup, as evidenced by greater absolute and relative reductions in mitral annular circumference (both *p* < 0.05).

Regarding mechanism, it should be noted that MitraClip is intended to reduce MR via device-induced leaflet coaptation - providing a nidus for increased leaflet stress and tension on peri-annular myocardium. In this context, our finding that MitraClip acutely reduced annular size supports the notion that tensile forces exerted by the device are sufficient to alter mitral apparatus geometry in regions beyond the clipped valve leaflets themselves, and that 3D TEE is sufficient to discern the remodeling effects of such tensile forces. Our finding of an association between pre-procedural dilation and MR recurrence also suggests that MitraClip-induced tensile forces may ultimately be insufficient in the context of marked annular or LV remodeling – a setting in which intrinsic resistance forces may be augmented, resulting in sub-optimal MitraClip response. While exact causality for MR recurrence cannot be discerned from our current results, it is also possible that MitraClip induced annular remodeling alters valve coaptation geometry, augments angular displacement between the mitral valves and sub-valvular apparatus, and/or induces myocardial tethering – each of which can contribute to MR. Consistent with the latter, recent computational modeling data from our group demonstrated that MitraClip augmented leaflet stress immediately adjacent to the device as well as LV myocardial stretch adjacent to the mitral annulus: [8] Our current results provide proof of concept that device-induced annular remodeling occurs in vivo, severity of which can be discerned on 3D TEE as a means of predicting therapeutic response to MitraClip. Whereas a large majority (70/80) of our study population underwent repair using the MitraClip NT/NTr device, our finding of an impact of MitraClip on annular remodeling is of relevance in context of new modifications of the device (XTr) that grasp a wider amount of leaflet tissue, and would thus be expected to apply a greater magnitude of force on the mitral annulus. It is also possible that greater magnitude of leaflet grasp would better resist tethering forces exerted by sub-valvular myocardium, and thus provide a more durable reduction of MR. In this context, future studies are warrented to test whether pattern and magnitude of induced annular remodeling are similar irrespective of MitraClip device type, and whether specific types of MitraClip mitigate or augment the impact of device-induced annular remodeling on post-procedure MR.

Our current results build upon a growing body of literature which have demonstrated chamber dilation to predict prognosis and therapeutic success among patients undergoing MitraClip. Large scale MitraClip registry data comprised primarily (91%) of patients with degenerative MR have also shown increased LV end-diastolic diameter to predict decreased likelihood of procedural success as assessed at time of hospital discharge [17]. Among patients with functional MR, differences in cardiac chamber remodeling may explain conflicting results of two recent MitraClip clinical trials (MITRA-FR, COAPT) [6, 7]. LV chamber size was over 25% larger in MITRA-FR vs. COAPT patients, paralleling a > 3-fold increase in recurrent severe MR: MITRA-FR reported MitraClip to have no impact on outcomes, whereas MR reduction in COAPT was accompanied by heart failure and mortality benefit. As further evidence of the importance of LV remodeling as a determinant of MitraClip response, prior research from our group (encompassing 67 patients included in the current cohort) showed increased LV size to be associated with risk for sub-optimal MitraClip response (>mild MR): [5] However, the relative utility of 2D and 3D derived mitral annular size independent of LV chamber volume, impact of the device on annular remodeling, as well as intra-procedural annular remodeling as a determinant of therapeutic response were not tested – providing key rationales for the current study.

Regarding imaging, our data highlight the utility of 3D echo as a pre-procedural planning tool to guide patient selection and predict therapeutic therapeutic response to MitraClip. Our results extend upon recent data showing mitral annular geometry on 3D TEE to stratify likelihood of MR reduction following MitraClip implantation. Among a cohort of 31 patients with degenerative MR, Oguz et al. reported that increased pre-procedural tenting height and volume on 3D TEE were each associated with risk for sub-optimal MR reduction [18]. Whereas our results found increased tenting height to be generally associated with suboptimal response, predictive value of this parameter was less (OR 1.17 per mm [CI 0.98–1.40], *p* = 0.09) than that of annular circumference or area (both *p* < 0.01). While reasons for this difference are uncertain, we speculate that it may stem from more advanced LV remodeling in our study population as evidenced by increased LV chamber size in our population (LV end diastolic diameter 6.0 ± 0.8 cm) compared to that studied by Oguz et al. (5.8 ± 0.7 cm): [18] Increased LV chamber size could potentially alter loading forces on the mitral valve, resulting in greater importance of pre-procedural mitral annular geometry as a determinant of MitraClip response. It is also possible that differences in valve pathology and/or myocardial substrate (i.e. fibrosis) could modify predictive utility of different mitral apparatus parameters. Given our sample size (*n* = 80) and the fact that device-induced annular remodeling is intrinsically related to pre-procedural chamber geometry, our analyses were insufficiently powered to test whether baseline LV chamber size and device-induced remodeling were independent predictors of MitraClip response, as well as whether pre-procedural LV geometry, myocardial substrate (fibrosis), and device-induced annular remodeling synergistically impacted MR recurrence after MitraClip. Further, larger scale, research is warranted to test the relative prognostic utility of valvular and annular remodeling indices – inclusive of device-induced changes in annular geometry - as predictors of MitraClip response.

Several limitations should be noted. First, our study defined optimal MitraClip response using a binary partition for MR (≤ mild [1+]), rather than a single quantitative measure. This approach is consistent with that employed in several prior studies in which greater severity of MR after MitraClip was shown to confer adverse prognosis [3, 4]. Despite this, a variety of cutoffs for adequate MR reduction have been used in prior MitraClip studies, which may explain variable rates of recurrent MR. [2–4, 19–22] Of note, procedural success among our cohort (41%) was near equivalent to that among degenerative MR patients undergoing MitraClip in the the EVEREST II trial, in which 43% of patients had ≤ mild MR at 1 year followup [2]. It is also important to recognize that follow-up was performed at a single time point after MitraClip, and that TTE was not performed immediately after device implantation. Accordingly, our analyses were unable to test relative contributions of residual MR and device-induced annular remodeling as determinants of MitraClip response – further studies employing serial imaging and more precise metrics of annular/myocardial stretch are necessary to expand on results of the current study. Finally, it should be noted that TEE was aquired using 3D technology, whereas TTE was limited to 2D. In this context, it is uncertain whether limits of TTE (i.e. lesser predictive utility of TTE quantified mitral annular size) stem from sub-optimal mitral annular visualization by TTE, or the 2D approach used for data aquistion. Given that TEE is invasive, further research is warranted to test whether 3D TTE provides equivalent utility for predicting MitraClip therapeutic response.

## Conclusions

This study demonstrates that MitraClip alters mitral annular geometry, and that magnitude of intra-procedural reduction in annular size is associated with sub-optimal device response (> mild MR). Future larger scale studies are warranted to further discern mechanism by which device-induced annular remodeling drives recurrent MR after MitraClip, and test clinical utility of 3D echo guided MR therapeutic strategies paired to pattern and magnitude of mitral apparatus remodeling.

## Data Availability

The data, analytic methods, and study materials will be made available to other researchers for purposes of reproducing the results or replicating the procedure, upon request (contingent on approval of the Weill Cornell Institutional Review Board and assurance of data de-identification).

## Abbreviations

2D: Two-dimensional
3D: Three-dimensional
CAD: Coronary artery disease
EROA: Effective regurgitant orifice area
LA: Left atrial
LV: Left ventricle; left ventricular
LVEF: Left ventricular ejection fraction
MI: Myocardial infarction
MR: Mitral regurgitation
PA: Pulmonary artery
TEE: Transesophageal echocardiography
TTE: Transthoracic echocardiography
